# Effectiveness of a physical activity program on cardiovascular disease risk in adult primary health-care users: the “Pas-a-Pas” community intervention trial

**DOI:** 10.1186/s12889-017-4485-3

**Published:** 2017-06-15

**Authors:** Victoria Arija, Felipe Villalobos, Roser Pedret, Angels Vinuesa, Mercé Timón, Teresa Basora, Dolors Aguas, Josep Basora, Pas-a-Pas research group, Eva Domínguez, Dolores Jovani, Gabriel Pascual, Lorenzo Peralta, Alicia Reche

**Affiliations:** 1 Unit of Research Support Reus-Tarragona, Institut d’Investigació en Atenció Primária, IDIAP Jordi Gol, Barcelona, Spain; 2 Faculty of Medicine and Health Sciences, Universitat Rovira i Virgili, Reus, Tarragona, Spain; 3 Institut d’Investigació Sanitària Pere Virgili, Reus, Tarragona, Spain; 4 Primary Health Care Area, Reus, Tarragona, Institut Català de la Salut, Generalitat de Catalunya, Barcelona, Spain; 5 Department of Activities and Projects, Reus Esport i Lleure SA, Reus, Tarragona, Spain; 6 Unit of Research Support Reus-Tarragona, Institut d’Investigació en Atencio Primária, (IDIAP) Jordi Gol (Barcelona), Camí de Riudoms 57, 43202 Reus, Spain

**Keywords:** Intervention program, Physical activity, Cardiovascular disease risk prevention, Primary care program

## Abstract

**Background:**

Physical activity is a major, modifiable, risk factor for cardiovascular disease (CVD) that contributes to the prevention and management of CVD.

The aim of this study was to assess the short- and medium-term effectiveness of 9 months of a supervised physical activity program, including sociocultural activities, on CVD risk in adults.

**Methods:**

Multicentered, randomized, controlled community intervention involving 364 patients in four primary care centers. The participants were randomly assigned to a Control Group (CG = 104) or Intervention Group (IG = 260); mean age 65.19 years; 76.8% women. The intervention consisted of 120 min/week walking (396 METs/min/week) and sociocultural gathering once a month. Clinical history, physical activity, dietary intake, CVD risk factors (smoking, systolic and diastolic blood pressure, weight, waist circumference, BMI, total cholesterol, LDL- and HDL-cholesterol, triglycerides, glycosylated hemoglobin and glucose) and global CVD risk were assessed at baseline and at the end of the intervention and multivariate models were applied to the data. Incidence of adverse cardiovascular events and continued adherence to the physical activity were assessed 2 years after intervention.

**Results:**

At the end of the intervention period, in the IG relative to the CG group, there was a significant increase in physical activity (774.81 METs/min/week), a significant change during the intervention period in systolic blood pressure (−6.63 mmHg), total cholesterol (−10.12 mg/dL) and LDL-cholesterol (−9.05 mg/dL) even after adjustment for potential confounders. At 2 years after the intervention, in the IG, compared with the CG, tthe incidence of adverse cardiovascular events was significantly lower (2.5% vs. 10.5%) and the adherence to regular physical activity was higher (72.8% vs 27.2%) in IG compared to CG.

**Conclusions:**

This community-based physical activity program improved cardiovascular health in the short- as well as medium-term, and promoted regular physical activity in the medium-term in older Spanish adults.

**Trials registration:**

Clinicaltrials.gov ID NCT02767739. Trial registered on May 5th, 2016**.** Retrospectively registered

## Background

Cardiovascular disease (CVD) is the leading cause of morbidity and mortality worldwide. For example, in 2012, 17.5 million people died from this cause and accounted for 31% of mortality. Further, the World Health Organization (WHO) estimates that by 2030, 23.3 million people will die from CVD [1].

Physical activity is a modifiable major risk factor for CVD [2–6]. The 2007 European Guidelines on Cardiovascular Disease Prevention in Clinical Practice recommended (as do other health-care organizations) at least 120 min/week of moderate physical activity [7] and, recently, the European Guidelines have increased the recommended levels of physical activity for adults [8]. Despite the health benefits of regular exercise, available data suggest that at least 31% of the world’s population is not meeting the minimum physical activity guidelines; the global prevalence of physical inactivity is 17% and, at 27.8%, is more prevalent in developed countries [9, 10].

Given the importance of physical activity in the control of CVD risk, the European Society of Cardiology, the European Association for Cardiovascular Prevention and Rehabilitation and the American College of Preventive Medicine have agreed a common policy statement to encourage integrated action by key stakeholders in order to achieve the broad adoption of a healthy lifestyle pattern of behavior (including physical activity) on a global scale.

Lifestyle interventions on CVD risk have been assessed in two previous cohort studies (both conducted in developed countries) and have shown an inverse relationship between physical activity and overall CVD risk. In a 7-year follow-up study of 23,747 Norwegian adults with no history of CVD, 2 sessions/week of moderate intensity physical activity reduced CVD risk by 49% [11]. A study conducted over 8 years in 41,675 Taiwanese adults found that those in the intervention group (at least 100 min of aerobic exercise/week) had a 14% lower CVD risk [12].

Several randomized controlled trials (RCT) have provided evidence of the benefits of physical activity on certain CVD risk factors, including reduction in systolic blood pressure (SBP) [13–15], improvements in lipid profile [16, 17], and anthropometric measurements [13, 18]. However, other studies found no such benefits [19–22].

These contradictory data could result from methodological differences in study design, target population, or type of physical activity used in the intervention program (frequency, duration, intensity, under supervision by health-care professionals). Of note is that none of these studies described differences in responses in relation to the population studied. For instance, these studies included individuals with or without CVD, individuals from primary care or general population, and individuals with different levels of existing physical activity. Also, with respect to duration of intervention, similar benefits were observed in interventions lasting from 4 to 12 months [13–18]. Further, greater benefits were observed in individuals performing 120 min/week moderate-intensity physical activity as those undertaking 60 min/week vigorous-intensity and supervised by health-care professionals and/or by a physical activity instructor [13–18] as compared with those who received physical activity advice alone [19, 20, 22, 23]. Of note is that none of these earlier RCTs assessed the medium-term effects of physical activity on CVD risk, and none analyzed the data using multivariate models adjusted for risk factors related to CVD.

Previous descriptive studies showed that inclusion of socio-cultural activities within a physical activity program was associated with increased social support networks and, consequently, with improved mental health and overall well-being which, in turn, promoted cardiovascular health quite apart from the physical activity benefit alone [24, 25].

In the light of these conflicting data, and based on current recommendations of physical activity [7, 26, 27] as well as previous physical activity interventions with proven benefits, the aim of the present study was to assess the short- and medium-term effectiveness of a 9-month supervised physical activity program, and including socio-cultural activities, on CVD risk in adults accessing primary health-care facilities.

## Methods

### Design

The study was multicentered, randomized, controlled, community intervention of a program of physical activity and socio-cultural activities intervention of 9 months duration The program was applied in adults drawn from among those attending 4 primary care centers (PCC) in the city of Reus (Catalonia; Spain). The incidence of CVD events was recorded at 2 years after the intervention. The Research Ethics Committee of the Institut d’Investigació en Atenció Primària de Salut (IDIAP), Jordi Gol, approved the study protocol. Data were analyzed in accordance with Consolidated Standards of Reporting Trials (CONSORT) guidelines for randomized trials. Clinicaltrials.gov ID NCT02767739.

### Participants and randomization

During a 6-month period prior to the intervention, all health-care professionals of the PCCs invited adult users of the services to participate in the program. Volunteers were directed to the nursing health-care professional of the PCC for an assessment of the individual’s eligibility criteria.

Inclusion criteria required the participant to be an adult accessing the PCC’s health-care facilities. Exclusion criteria were: an episode of ischemic heart disease (<6 months previously), or an acute episode of arthritis which would limit the ability to walk, or having a lung or heart disease with dyspnea (mild to moderate effort dyspnea) which would limit the individual’s ability to undertake the proposed exercise regimen.

Participants who met the eligibility criteria, signed the informed consent document and were individually randomized to the IG or CG in a ratio of 3:1 using a table of computer generated random numbers.

The sample size calculation was based on global CVD risk (REGICOR) as the main dependent variable, and using the following criteria: an alpha risk of 0.05 and a beta risk of 0.2 in a bilateral contrast. Assuming a patient loss-to-follow-up of 10%, a standard deviation of 4% and a difference of ≥1.5 units, 250 participants in the IG and 82 in the CG were needed. The estimated sample size was calculated using the Granmo software (version 7.12; Granmo; IMIM Hospital del Mar, Barcelona, Spain).

### Intervention

Based on physical activity recommendations [7, 26, 27], the intervention program consisted of supervised walking (396 METs/min/week over 120 min in 2 walking sessions per week of 60 min each) and socio-cultural activities once a month. A physical activity specialist was responsible for the standardization of procedures and for the training of the primary care nurses. Walking itineraries and cultural activities were pre-set. Walking itineraries were, on average, a five-kilometer circuit in and around the city. Group sizes ranged from 15 to 30 participants. Monthly socio-cultural activities included: visits to museums and libraries, cultural exhibitions, tourist attractions and dance lessons.

The program was supervised by the health-care professionals who accompanied the participants in all the activities, and who closely monitored the participants’ adherence to the program.

Participants who were randomized to CG received usual care from health-care personnel, and were recommended to follow their habitual lifestyle.

## Measurements

### Outcomes measured at baseline

#### Clinical history

All participants had the following diagnoses recorded: hypertension, type 2 diabetes, dyslipidemia, overweight, obesity, depression, anxiety and osteoporosis.

#### Socio-demographic characteristics

Age, sex, social class, and educational level were obtained from clinical data and face-to-face interviews. Class status was assessed using an adaptation of the British Registrar General classification which yields three class categories: high (class I-II), middle (class III_N_-III_M_) and lower (class IV-V) [28].

### Outcomes measured at baseline and at the end of the intervention

#### Cardiovascular disease risk assessment

Blood pressure was measured with a manual sphygmomanometer with the participants resting for at least five minutes. Three recordings were taken and the average of the second and third readings was used in the statistical analyses. The CVD risk was estimated using the scale “Registre Gironí del Cor” (REGICOR), based on Framingham criteria standardized for the Spanish population. This scale includes sex, age, diabetes (yes, no), smoking (yes, no), systolic and diastolic blood pressure and serum cholesterol levels [29]**.**

#### Physical activity

Levels were measured using the short version of the International Physical Activity Questionnaire (IPAQ-S) validated for the Catalan population [30]. The variables such as intensity (light, moderate, or vigorous), frequency and duration of physical activity in the previous 7 days, were collected using the IPAQ-S. The frequency and intensity of each activity was used to calculate the total of an intensity category in terms of METs/min/week. These values were obtained by multiplying the average energy expenditure (3.3 MET for walking, 4.0 MET for moderate intensity, and 8.0 MET for vigorous intensity) by min/week for each physical activity. The results of each category of activity intensity were summed to obtain the total physical activity in METs/min/week. Based on total physical activity, participants were classified into 3 levels of physical activity: low (<600 METs/min/week), moderate (≥600–2999 METs/min/week) and high (≥3000 METs/min/week).

#### Frequency of food consumption

Food consumption was assessed using a validated food frequency questionnaire containing 45 items [31]. A face-to-face interview was conducted in the PCC setting by a nurse, who recorded times per week and times per month for each food ration consumed and the rations consumed per day were calculated. To calculate g/day of each food item, daily portions were multiplied by grams of each item consumed relative to reference data of food consumption evaluated in the same population and established by expert nutritionists [32]. Foods were grouped according to their nutrient composition: dairy (milk, yogurt, dairy desserts, cheese); meat/fish/eggs (red, white, processed meat and cold meat, lean, fatty fish and shellfish); salad cereals (rice, pasta, bread, legumes and potatoes); sweetened cereals (pastries, biscuits, breakfast cereals); fruits/vegetables (salad, tomatoes, vegetables side dish, aubergines, courgettes, mushrooms; green beans, chards, spinach, fresh fruit and canned fruit); oils; nuts; and beverages (fermented beverages).

#### Anthropometric measurements

Weight (kg) was measured using a calibrated balance with the measurements taken to the nearest 0.1 kg. Height (cm) was measured using a calibrated balance with the measurements taken to the nearest 1 cm. Weight and height measurements were then used to calculate the body mass index [BMI as kg/m^2^]. Waist circumference was measured at the top of the hip bone below the ribs with the tape measure placed in the middle between these points, and wrapped around the waist.

#### Biochemical analysis

The biochemical parameters analyzed were triglycerides, total cholesterol, HDL- and LDL-cholesterol, glycated hemoglobin (HbA1c) and glucose. A study profile for each individual was generated using all these parameters at the Tarraco laboratory (ISO9001:2000 certified ICS Tarragona laboratory). Fasting blood was extracted at the PCC by the primary-care nurses and transported on ice to the central laboratory for analyses in as short a time-lapse as possible.

#### Changes during intervention

Changes during the intervention period with respect to food consumption, anthropometric measures and CVD risk factors and the estimated REGICOR score were calculated as the difference between the end-of-program and the baseline values. In addition, the change in physical activity levels in the participants by the end of the intervention period (compared with baseline levels) was calculated. The participants were, then, classified into 3 categories: lower, similar (unchanged) or higher.

#### Adverse cardiovascular events and adherence to physical activity 2 years after the intervention

The assessment of the incidence of adverse cardiovascular events such as acute myocardial infarction (AMI) and cerebrovascular accident (CVA) 2 years after the intervention, was calculated using the computerized clinical histories maintained at the PCC where the participants attended, and the databases of the hospitals where the adverse events were treated.

To assess the adherence to physical activity, the participants were contacted by telephone and were asked if they had continued the physical activity along the lines of the intervention characteristics.

#### Statistical analysis

All categorical variables were described as percentages while means and standard deviation were reported for continuous variables. The χ^2^ test was used to compare categorical variables in different groups. Unpaired Student’s *t*-test was used to compare continuous variables, while the paired Student *t*-test was used to compare values between different time-points (baseline and at the end of the intervention) for continuous variables, while the McNemar test was used for the categorical variables.

Multiple linear regression models were applied to assess the effect of the intervention (0, 1) as an independent variable, with CVD risk factors as dependent variables (SBP, DBP, weight, BMI, waist circumference, total cholesterol, LDL- and HDL-cholesterol, triglycerides, and glucose) and CVD risk evaluated on the REGICOR scale. The following covariates were considered: age (years), sex (male, female), social class (dummy variables comparing social class were created; low (reference) versus middle and high); PCC (dummy variables comparing centers were created; PCC1 (reference) versus PCC2, PCC3, PCC4), smoking (no, yes), BMI (kg/m^2^), diagnoses at baseline: hypertension (no, yes), type 2 diabetes (no, yes), dyslipidemia (no, yes), depression (no, yes), anxiety (no, yes), osteoporosis (no, yes) and the dependent variable of each model at baseline.

Poisson regression was used to assess the relationship between physical activity and adverse CVD events in participants 2 years after the intervention. In this assessment, the number of adeverse CVD events was considered the dependent variable while the intervention (0, 1) as the independent variable. This model was adjusted for sex (male/female), diagnosis of systemic arterial hypertension, dyslipidemia, overweight, obesity, anxiety, depression, and changes during intervention (end vs. baseline) of the values of the following variables: systolic arterial pressure, total cholesterol and LDL-cholesterol. The coefficient β and the standard error, obtained in the Poisson regression, were used to calculate the relative risk with the 95% confidence interval.

The results were analyzed as per protocol (PP). The intention-to-treat (ITT) analyses were also performed to verify the consistency between the results of both types of analysis, as follows: an analysis of sensitivity was performed in which lost values were imputed using multiple imputation (MI) from linear regression models in which 5 different combinations of data were created. For the MI we used the following predictive variables: age, gender, social class, diagnosis of chronic illness and change in the CVD indicators during the course of the intervention.

Statistical significance was set at *p* value <0.05. The statistical software SPSS for Windows Version 22.0 (SPSS Statistics 22.0) was used throughout.

## Results

**Fig. 1:**
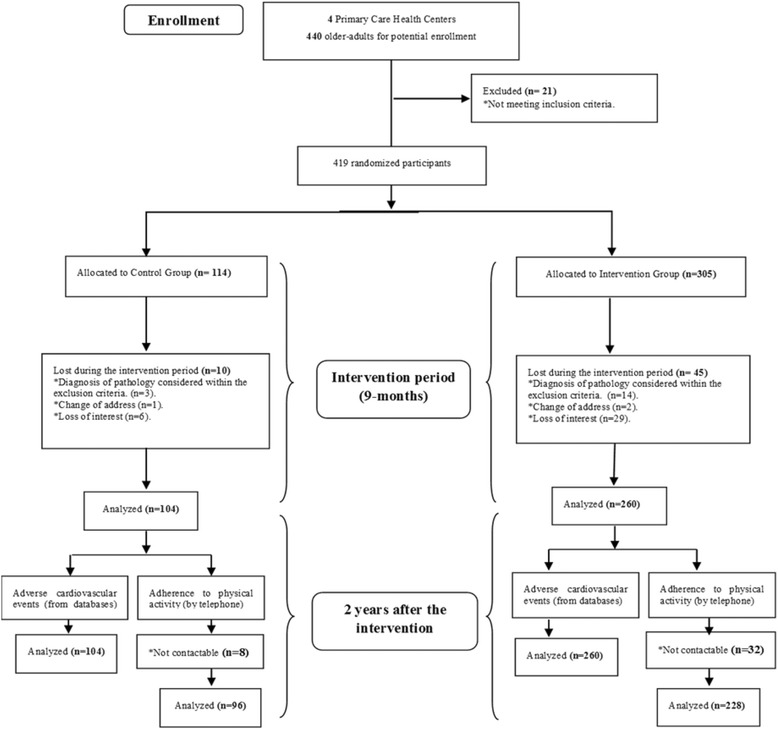
Flow diagram of the study

There were 419 participants recruited from 4 PCC and assigned to CG (*n* = 114) or IG (*n* = 305). During the intervention period 13% dropped out: 8.8% belonging to the CG and 14.7% to the IG. Dropout causes were: diagnosis of a pathology considered within the exclusion criteria (29.1%); change of address (3.6%) and loss of interest by the participant (67.3%). By the end of the intervention period, 364 participants had completed the program (87%). Two years after the intervention, participants were contacted by telephone to determine the incidence of adverse cardiovascular events and adherence to the physical activity recommendations. In total, 96 participants of CG (92%) and 228 of the IG (91.5%) were contacted (Fig. 1)

There were no statistically significant differences with respect to age, gender, social class and the presence of chronic medical conditions (*p* > 0.05) between the participants who dropped-out compared to those who continued in the program.

There were no significant differences in the socio-demographic characteristics, social class, and risk factors between groups at baseline (Table 1). Neither were there differences in the changes during intervention in food item consumption during the trial period between CG and IG, except in the food group of the sweetened cereals (Table 2).

**Table 1:** Baseline characteristics of the participants in the control and intervention groups

	Control group	Intervention group	*p*
	(*n* = 104)	(*n* = 260)	
Age (years)^a^	66.99 (10.28)	64.5 (9.20)	0.400
Women (%)	71.90	78.70	0.116
Social Class			
High Class I-II (%)	27.08	23.70	0.222
Middle Class III_N_-III_M_ (%)	59.30	54.50	
Lower Class IV-V (%)	13.54	21.70	
CVD risk factors			
Smoking (%)	4.20	7.90	0.123
Hypertension (%)	57.30	54.20	0.343
Type 2 diabetes (%)	18.80	20.60	0.766
Overweight (%)	37.50	39.10	0.807
Obesity (%)	46.90	42.70	0.546
Dyslipidemia (%)	50.00	49.40	1.000
Depression (%)	10.40	16.20	0.234
Anxiety (%)	24.00	19.00	0.301
Without contributing pathology (%)	5.20	6.30	0.458

^a^Values expressed as mean and (standard deviation)

**Table 2:** Food item consumption at baseline, and changes during intervention

Food Consumption^a^	Baseline		*p*	Changes during intervention (End - Baseline)		*p*
	CG (*n* = 104)	IG (*n* = 260)		CG (*n* = 104)	IG (*n* = 260)	
Dairy Products (g/day)	378.50 (185.80)	325.21 (142.85)	0.007	9.76 (171.49)	14.13 (122.04)	0.823
Meat/Fish/ Eggs (g/day)	148.87 (53.60)	147.59 (56,61)	0.857	2.40 (53.98)	6.69 (63.26)	0.569
Salad Cereals (g/day)	124.86 (59.76)	124.87 (51.29)	0.998	3.42 (65.09)	−5.23 (52.62)	0.223
Sweetened Cereals (g/day)	28.20 (36.51)	21.47 (26.49)	0.073	−8.60 (25.65)	−1.80 (25.43)	0.033
Fruits/ Vegetables (g/day)	328.59 (142.02)	302.98 (143.81)	0.150	−13.34 (150.78)	13.28 (146.53)	0.149
Nuts (g/day)	4.81 (6.92)	4.00 (5.09)	0.256	−0.96 (6.64)	0.21 (5.34)	0.148
Beverage (g/day)	55.32 (82.32)	53.57 (81.97)	0.864	8.27 (82.95)	−5.61 (89.12)	0.201

^a^Values expressed as mean and (standard deviation)

*CG* Control group, *IG* Intervention group

In the intervention group, total physical activity significantly increased during intervention (774.81 METs/min/week) whereas it decreased in the control group (−357.61 METs/min/week) (Table 3). In addition, in the IG levels of physical activity significantly increased during the intervention, compared to the control group (*p* = 0.033). Walking every week accounted for 396 METs/min/week of physical activity energy expenditure. Average attendance at walking sessions was 74.3%, which represents a mean expenditure of the participant group of 303.6 METs/min/week.

**Table 3:** Physical activity at baseline, and changes during intervention

	Baseline				Change during the intervention (End - Baseline)		
	CG (*n* = 104)	IG (*n* = 260)	*p*		CG (*n* = 104)	IG (*n* = 260)	*p*
Total physical activity (METs/min/week)^a^	2468.26 (4628.84)	2363.10 (3122.32)	0.808		−357.61 (4765.03)	774.81 (4004.22)	0.026
Level of physical activity					% change		
Low (%)	32.30	28.00	0.610	Lower	61.5	45.8	0.033
Moderate (%)	46.90	46.80		Similar	26.0	39.0	
High (%)	20.80	25.20		Higher	12.5	15.3	

*CG* Control group, *IG* Intervention group, *METs* Metabolic equivalents

^a^Values expressed as mean and (standard deviation)

CVD risk indicators in the trial population are shown in Table 4. At baseline there were no significant differences between the control and intervention groups. However, there were significant differences with respect to change during intervention in systolic blood pressure (−3.59 mmHg), total cholesterol (−10.68 mg/dL) and LDL-cholesterol (−7.18 mg/dL) levels in the IG vs. the CG.

**Table 4:** CVD risk inicators at baseline, and changes during intervention

	Baseline			Changes during intervention (End - Baseline)		
	CG (*n* = 104)	IG (*n* = 260)	*p*	CG (*n* = 104)	IG (*n* = 260)	*p*
SBP (mmHg)	135.32 (16.62)	131.06 (15.94)	0.280	0.90 (18.63)	−3.59 (16.45)	0.029
DBP (mmHg)	75.96 (9.86)	76.75 (9.09)	0.478	−1.29 (10.64)	−3.03 (9.75)	0.140
Weight (Kg)	75.72 (14.92)	75.37 (14.80)	0.843	0.08 (4.52)	−1.23 (8.02)	0.151
BMI (Kg/m^2^)	29.95 (4.88)	30.01 (5.02)	0.924	−0.03 (1.91)	−0.22 (2.38)	0.471
WC (cm)	100.31 (11.74)	100.50 (11.81)	0.895	−1.58 (5.79)	−2.96 (8.99)	0.168
Total Cholesterol (mg/dL)	206.83 (32.67)	205.26 (36.58)	0.713	0.09 (32.96)	−10.68 (31.81)	0.006
HDL-cholesterol (mg/dL)	56.37 (16.09)	55.51 (14.86)	0.641	−1.18 (10.26)	−0.07 (12.37)	0.435
LDL-cholesterol (mg/dL)	122.70 (29.62)	122.33 (34.14)	0.922	1.82 (26.48)	−7.18 (29.04)	0.009
TG (mg/dL)	134.63 (83.91)	132.26 (67.75)	0.786	−3.11 (60.33)	−3.26 (60.26)	0.983
HbA1c (%)	7.21 (1.61)	7.40 (1.37)	0.599	−0.33 (1.42)	−0.08 (1.20)	0.476
Glucose (mg/dL)	99.19 (33.91)	98.66 (28.42)	0.883	−5.16 (21.04)	2.96 (19.45)	0.001
REGICOR	4.63 (3.54)	4.60 (3.97)	0.962	0.37 (2.53)	−0.28 (2.55)	0.064
Change from smoking to non-smoking						
Smoking (%)	4.20	7.90	0.123	1.00	0.90	0.369

Values are expressed in mean and (standard deviation)

*CG* Control group, *IG* Intervention group, *SBP* Systolic blood pressure, *DBP* Diastolic blood pressure, *BMI* Body mass index, *WC* Waist circumference, *HDL* High-density lipoprotein, *LDL* Low-density lipoprotein, *TG* Triglycerides, *HbA1c* Glycated hemoglobin; Cardiovascular disease risk REGICOR scale

Two years after intervention the number of adverse cardiovascular events was significantly lower in the IG (5 cases: 2 AMI, 3 CVA) compared to the CG (10 cases: 8 AMI, 2 CVA); with a relative risk of 0.15 (95% CI 0.04–0.51). With respect to adherence to physical activity, 72.8% of subjects in the IG continued performing physical activity with similar characteristics to that of the intervention, compared with 27.2% of the CG (*p* = 0.010).

Table 5 summarizes the effects of the physical activity intervention on CVD risk factors, and the REGICOR scale. There were beneficial effects of the intervention on SBP (−6.63 mmHg) total cholesterol (−10.12 mg/dL), LDL-cholesterol (−9.05 mg/dL) and the REGICOR score (−0.72%).

**Table 5:** Effects of the physical activity intervention program on CVD risk factors, and the REGICOR scale

	β	SE	*P*	
Model 1 SBP (mmHg)				
Intervention (control, intervention)	−6.63	1.67	0.001	R^2^_c_ × 100 = 31.9%
Age (years)	0.23	0.10	0.024	F_17,331_ = 9.1
Hypertension (no, yes)	3.91	0.12	0.015	*p* < 0.001
Dyslipidemia (no,yes)	3.06	1.51	0.045	
Smoking (no, yes)	7.17	2.87	0.013	
BMI (kg/m^2^)	0.42	0.15	0.001	
Baseline SBP (mmHg)	0.30	0.46	0.006	
Model 2 Cholesterol (mg/dL)				
Intervention (control, intervention)	−10.12	3.48	0.004	R^2^_c_ × 100 = 42.2%
Social Class (low; middle)	−10.65	4.53	0.020	F_17,328_ = 14.0
Social Class (low, high)	−14.03	4.87	0.004	*p* < 0.001
Type 2 diabetes (no, yes)	12.89	3.85	0.001	
Anxiety (no, yes)	8.21	4.15	0.049	
Baseline cholesterol (mg/dL)	0.55	0.47	0.001	
Model 3 LDL-cholesterol (mg/dL)				
Intervention (control, intervention)	−9.05	3.07	0.003	R^2^_c_ × 100 = 41.9%
Type 2 diabetes (no, yes)	9.11	3.43	0.008	F_17, 329_ = 36.8
Baseline LDL-cholesterol	−0.59	0.04	0.001	*p* < 0.001
Model 4 Glucose (mg/dL)				
Intervention (control, intervention)	8.21	2.29	0.001	R^2^_c_ × 100 = 65.8%
Type 2 diabetes (no, yes)	19.90	3.32	0.001	F_17, 326_ = 36.8
Baseline Glucose (mg/dL)	0.57	0.04	0.001	*p* < 0.001
Model 5 REGICOR (%)				
Intervention (control, intervention)	−0.72	0.32	0.028	R^2^_c_ X 100 = 60.3%
Sex (men, women)	−1.19	0.35	0.001	F_17,260_ = 23.2
Baseline REGICOR	-0.57	0.04	0.001	*p* < 0.001

Multiple linear regression models adjusted for intervention group (0 = control group; 1 = Intervention group); age (years), sex (0 = men; 1 = women); Social Class (dummy variables, 0 = reference); 4 Primary Care Centers (dummy variables, 0 = reference), smoking (0 = no; 1 = yes); BMI (kg/m^2^), hypertension (0 = no; 1 = yes); type 2 diabetes (0 = no; 1 = yes); dyslipidemia (0 = no, 1 = yes), depression (0 = no; 1 = yes); anxiety (0 = no; 1 = yes); osteoporosis (0 = no; 1 = yes); and the dependent variable of each model at baseline. Only significant variables are shown

No significant effects of intervention on DBP, weight, waist circumference, BMI, triglycerides and HDL-cholesterol, and HbA1c dependent variables were observed.

The results are presented as per protocol analysis (PP). The results of the ITT analysis were similar to the PP results of the intervention with respect to all the CVD risk factors measured.

## Discussion

This community-based, randomized, controlled, intervention program among adults attending primary care clinics, has demonstrated that implementing a program to promote physical activity over a period of 9 months significantly decreased SBP, total and LDL-cholesterol levels and overall CVD risk. The results were analyzed using multivariate techniques to adjust for confounding variables associated with the putative causes. These included: socio-demographic, lifestyle, morbidity and anthropometric measures. There were no differences in the dietary intake of participants during the intervention and, as such, the effects on CVD appear specific to the physical activity intervention i.e. related to the ability of the intervention program to decrease SBP and to improve lipid profile. Further, the present study led to an important reduction of major adverse cardiovascular events assessed 2 years after the intervention. Importantly, our results demonstrated that the implementation of an interactive health-education strategy improves CVD risk outcomes in our older Spanish adults.

Conducting a RCT, and controlling the risk factors associated with the health outcomes studied, highlighted the evidence of the impact of a physical activity intervention on CVD risk. In our study a large number of participants were randomized to the IG, in a 3:1 ratio, since the favorable effect of the intervention was predictable (based on published literature) and, as such, would benefit a greater number of individuals. We also took into account the expected high drop-out rate in the IG during follow-up.

General and clinical characteristics of study subjects are similar to previous interventions with respect to age, socio-economic status, level of education and the prevalence of chronic diseases and conditions [13–18].

At the time of the study design, participants were instructed to perform 120 min/week of moderate-intensity physical activity based on global recommendations [7, 26, 27] although more recent European guidelines propose 150 min/week of moderate-intensity physical activity [8]. We also took into account the characteristics of previous physical activity intervention programs that showed major benefits on the individuals’ outcomes i.e. health benefits of physical activity intervention are higher when the intervention is supervised by health-care personnel and/or a physical activity professional [13, 16–18]. In addition, as has been demonstrated, the inclusion of socio-cultural activities in the intervention program increases a person’s well-being and improves mental health; both of which are related to decreased CVD risk [24, 33].

The strengths of our study include the assessment of physical activity using a validated questionnaire [30], the evaluation of CVD risk factors and food consumption at two time-points (at baseline and at the end of the 9 month active intervention). Since diet is one of the main modifiable CVD risk factors, we assessed food consumption pre- and post-intervention using a food-frequency questionnaire previously validated in our population [31].

In addition, we assessed a wide range of CVD risk factors, as recommended by the European Society of Cardiology. These included smoking, blood pressure, weight, waist circumference, BMI, total cholesterol, LDL- and HDL- cholesterol, triglycerides, glycated hemoglobin and glucose. Also, a novelty of the present study was that a global CVD risk and incidence of adverse cardiovascular events 2 years after the intervention were assessed in order to have a broader overview of the effect of physical activity on all CVD risk factors. To estimate the CVD risk we used the REGICOR scale. This scale is the most recommended for primary prevention of coronary heart disease in Spain because other methods such as the SCORE or Framingham scales overestimate the individual’s CVD risk [34].

Our results showed that physical activity energy expenditure increased by 774.81METs/min/week (24%) in the IG between baseline and the end of the intervention. This was more than double that achieved in the intervention program (303.6 METs/min/week). The energy expenditure of 774.81 METs/min/week is equivalent to a physical activity time of 284.86 min/week. This physical activity time is even higher than the revised level proposed by the new European Guidelines on Cardiovascular Disease Prevention in Clinical Practice (150 min/week of moderate physical activity) [8]. Conversely, total physical activity decreased by 357.61 METs/min/week in the CG by the end of the intervention period, relative to baseline. These data clearly indicate the effectiveness of the intervention program in promoting physical activity. Other RCTs employing a 12-month supervised physical activity program over 90–120 min/week showed an increase of 15 to 27% in total physical activity [13, 15–18], and is concordant with our study (24%) findings. In contrast those programs based on physical activity advice alone, observed a minor increase in total physical activity [19, 20, 22, 23]. In addition, our program not only encouraged activity in excess of the physical activity of the intervention program but also this beneficial habit was seen to be consolidated in a large percentage of participants 2 years after the intervention.

With respect to improvement in CVD risk factors, we observed a decrease in SBP (−6.63 mmHg), total cholesterol (−10.12 mg/dL), and LDL-cholesterol (−9.05 mg/dL). Overall CVD risk was reduced (−0.72%), based on multivariate models adjusted for variables associated with the putative causes of CVD. For example, a reduction in SBP of 3 mmHg decreases peripheral vascular resistance [35], since physical activity increases the concentrations of nitric oxide resulting in arterial vasodilation leading to decreases in peripheral vascular resistance and increased blood perfusion [4]. This effect is enhanced when there is a decrease in LDL-cholesterol levels and an increase in HDL-cholesterol levels, the latter (HDL-cholesterol) modulating the synthesis of nitric oxide in the endothelium [36].

The findings of this study are concordant with the results of another RCT. As in our study, Halber, et al. found a benefit of physical activity on CVD risk factors in Australian users of primary care facilities. They observed a decrease of 21 mg/dL in total cholesterol and 13.99 mg/dL in LDL-cholesterol levels. However, no effect was observed on other variables assessed: SBP, DBP, weight, triglycerides and HDL-cholesterol [16]. Although the reduction of LDL-cholesterol levels in our intervention was a mean of 8.76 mg/dL, evidence from previous studies indicate that a reduction of only 2 mmol/L (7.69 mg/dL) in LDL-cholesterol levels was effective in reducing the formation of atherosclerotic plaques [36].

Other similar RCTs had obtained different effects on the CVD risk factors studied. Kim et al. observed, in sedentary Japanese, a decrease of 31.2 mg/dL in triglyceride levels, a reduction of 1.47 cm in waist circumference, and an increase of 12.17 mg/dL in HDL-cholesterol levels. However, they did not find any significant effect on SBP, DBP and glucose [17]. Anderson et al., in a study of Pakistani immigrants in Norway, observed a reduction of 1.9 cm in waist circumference; but no significant changes during intervention in SBP, DBP, triglycerides, total cholesterol and LDL- or HDL- cholesterol [18].

Earlier RCT studies examining the impact of shorter physical activity programs than the current recommended guidelines, found little or no beneficial effects on CVD factors. Studies in sedentary English and African-American participants enrolled in a supervised physical activity program including less aerobic activities (45–90 min/week) than ours, observed a higher decrease in the individual’s SBP (5–12 mmHg) than that observed in our study. However, no significant benefits on other CVD risk variables were observed [14, 15]. It is of note that other RCTs found no beneficial effects of a physical activity program on CVD risk factors, probably because the intervention was based on providing advice alone (educational materials and follow-up medical visits) [19, 20, 22, 23].

Despite the beneficial impact on health outcomes observed, our intervention also led to an increase of glucose levels in the IG (2.96 mg/dL). This could be explained, in part, by the duration and type of aerobic exercise in our intervention. The guidelines for better glycemic control proposed by the American Diabetics Association (ADA) recommends the performance of 150 min/week of moderate-vigorous physical activity [37]. In addition, the ADA suggests (as do some studies) performing combined aerobic and resistance exercises because of better effects on insulin sensitivity and in glycemic reduction, compared to performing only one type of exercise [38].

With regard to overall CVD risk, few RCTs have observed decreased CVD risk following a physical activity intervention program. Tiessen, et al., studied Dutch patients accessing primary care facilities. The intervention was a 12-month program consisting on 20 min physical activity advice sessions per month. [24]. They observed a decrease of 1.8% in CVD risk, which was higher than that observed in our study (−0.72%), However, Garcia-Ortiz, et al., found no effect on similar Spanish participants following a physical activity program with similar characteristics to the Dutch study [20].

Our intervention has mid-term beneficial effects on adverse cardiovascular events. We observed a lower percentage of these events in the IG compared to the CG (2.5% vs. 10.4%) 2 years after the intervention. Other observational studies have demonstrated an inverse relationship between physical activity and the risk of adverse cardiovascular events. In a cohort study of 6213 sedentary American adults with follow-up over 13 years, >50% reduction in mortality risk was observed in those individuals with a higher level of physical activity [39]. In the Women’s Health Study of 10.9 years follow-up, the risk of CVD decreased in relation to higher levels of physical activity, together with a reduction in adverse cardiovascular events [40].

Despite the outcomes observed in previous physical activity intervention programs being very variable (due, in part, to the characteristics of the program or the population studied), most of the studies found at least some beneficial effects on CVD risk factors and, as such, provides support for intervention programs within the community setting. Hence, more studies are needed to confirm the beneficial effects on cardiovascular health based on reliable information and outcomes.

## Conclusion

The physical activity program supervised by health-care personnel, and including socio-cultural activities, aimed at adults drawn from primary care facilities improved cardiovascular disease risk by the end of the intervention period of 9 months. The beneficial changes observed during intervention were in SPB, total cholesterol and LDL-cholesterol, and overall CVD risk score. These outcomes were independent of the participant’s food intake during the intervention program and, as well, other factors related to CVD such as socio-demographic factors, lifestyle and the presence of disease. Two years after the intervention, the intervention group developed a lower incidence of adverse cardiovascular events and a higher adherence and continuation of physical activity, compared to the CG. The broad benefit in terms of cardiovascular benefit obtained from the intervention program was, in part, possibly due to the characteristics of the program i.e. being supervised by health-care personnel and the inclusion of group socio-cultural activities. The promotion and support of physical activity should be a global priority in primary care because of the health benefits accruing to adults participating in such programs.

## Abbreviations

ADA: American Diabetics Association
BMI: Body mass index
CG: Control group
CVD: Cardiovascular diseases
DBP: Diastolic blood pressure
HbA1c: Hemoglobin A1c
HDL-cholesterol: High density lipoprotein cholesterol
IG: Intervention group
IPAQ-S: International physical activity questionnaire, short version
LDL-cholesterol: Low density lipoprotein cholesterol
METs: Metabolic equivalents
NO: Nitric oxide
PCC: Primary care center
RCT: Randomized controlled trial
SBP: Systolic blood pressure
TG: Triglycerides
WC: Waist circumference
WHO: World health organization.

## References

[1] Organizacion Mundial de la Salud. Informe sobre la situación mundial de las enfermedades no transmisibles. WHO. 2014;1–18.

[2] Claas SA, Arnett DK (2016). The role of healthy lifestyle in the primordial prevention of cardiovascular disease. Curr Cardiol Rep.

[3] Truthmann J, Busch MA, Scheidt-Nave C, Mensink GBM, Gößwald A, Endres M (2015). Modifiable cardiovascular risk factors in adults aged 40-79 years in Germany with and without prior coronary heart disease or stroke. BMC Public Health.

[4] Schuler G, Adams V, Goto Y (2013). Role of exercise in the prevention of cardiovascular disease: results, mechanisms, and new perspectives. Eur Heart J.

[5] Dalleck LC, Van Guilder GP, Quinn EM, Bredle DL (2013). Primary prevention of metabolic syndrome in the community using an evidence-based exercise program. Prev. Med. (Baltim).

[6] Stensvold D, Nauman J, Nilsen TIL, Wisløff U, Slørdahl SA, Vatten L (2011). Even low level of physical activity is associated with reduced mortality among people with metabolic syndrome, a population based study (the HUNT 2 study, Norway). BMC Med. BioMed Central.

[7] Guía Europea de Prevención Cardiovascular en la Práctica Clínica (2009). Adaptación española del CEIPC 2008 * Comité Español Interdisciplinario para la Prevención Cardiovascular. Rev. Clin. Española.

[8] Piepoli MF, Hoes AW, Agewall S, Albus C, Brotons C, Catapano AL (2016). 2016 European guidelines on cardiovascular disease prevention in clinical practice. Eur Heart J.

[9] Dumith SC, Hallal PC, Reis RS, Kohl HW (2011). Worldwide prevalence of physical inactivity and its association with human development index in 76 countries. Prev Med (Baltim).

[10] Kohl HW, Craig CL, Lambert EV, Inoue S, Alkandari JR, Leetongin G (2012). The pandemic of physical inactivity: global action for public health. Lancet.

[11] Hamer M, Stamatakis E (2012). Low-dose physical activity attenuates cardiovascular disease mortality in men and women with clustered metabolic risk factors. Circ Cardiovasc Qual Outcomes.

[12] Wen CP, Wai JPM, Tsai MK, Yang YC, Cheng TYD, Lee M-C (2011). Minimum amount of physical activity for reduced mortality and extended life expectancy: a prospective cohort study. Lancet.

[13] Salinas CJ, Bello SM, Flores CA, Carbullanca LL, Torres GM (2005). Actividad física integral con adultos y adultos mayores en Chile: Resultados de un programa piloto. Rev. Chil. Nutr. Sociedad Chilena de Nutrición, Bromatología y Toxicología.

[14] Murphy MH, Murtagh EM, Boreham CA, Hare LG, Nevill AM (2006). The effect of a worksite based walking programme on cardiovascular risk in previously sedentary civil servants. BMC Public Health.

[15] Duru OK, Sarkisian CA, Leng M, Mangione CM (2010). Sisters in motion: a randomized controlled trial of a faith-based physical activity intervention. J Am Geriatr Soc.

[16] Halbert J, Silagy C, Finucane P (2000). Physical activity and cardiovascular risk factors: effect of advice from an exercise specialist in Australian general practice. Med J Aust.

[17] Kim J, Tanabe K, Yoshizawa Y, Yokoyama N, Suga Y, Kuno S (2013). Lifestyle-based physical activity intervention for one year improves metabolic syndrome in overweight male employees. Tohoku J Exp Med.

[18] Andersen E, Høstmark AT, Anderssen SA (2012). Effect of a physical activity intervention on the metabolic syndrome in Pakistani immigrant men: a randomized controlled trial. J Immigr Minor Health.

[19] Elley CR, Kerse N, Arroll B, Robinson E (2003). Effectiveness of counselling patients on physical activity in general practice: cluster randomised controlled trial. BMJ.

[20] García-Ortiz L, Grandes G, Sánchez-Pérez Á, Montoya I, Iglesias-Valiente JA. Recio-Rodríguez JI, et al. Efecto en el riesgo cardiovascular de una intervención para la promoción del ejercicio físico en sujetos sedentarios por el médico de familia. Rev. Española Cardiol. 2010;63:1244–1252.

[21] Liira H, Engberg E, Leppävuori J, From S, Kautiainen H, Liira J (2014). Exercise intervention and health checks for middle-aged men with elevated cardiovascular risk: a randomized controlled trial. Scand. J. Prim. Health Care.

[22] Lawton BA, Rose SB, Elley CR, Dowell AC, Fenton A, Moyes SA (2008). Exercise on prescription for women aged 40-74 recruited through primary care: two year randomised controlled trial. BMJ.

[23] Van Sluijs EMF, Twisk JWR, Calfas KJ, van Poppel MNM, Chin A, Paw MJ (2005). Effect of a tailored physical activity intervention delivered in general practice settings: results of a randomized controlled trial. Am J Public Health.

[24] Tiessen AH, Smit AJ, Broer J, Groenier KH, van der Meer K (2012). Randomized controlled trial on cardiovascular risk management by practice nurses supported by self-monitoring in primary care. BMC Fam Pract.

[25] Kouvonen A, De Vogli R, Stafford M, Shipley MJ, Marmot MG, Cox T (2012). Social support and the likelihood of maintaining and improving levels of physical activity: the Whitehall II study. Eur J Pub Health.

[26] Haskell WL, Lee I-M, Pate RR, Powell KE, Blair SN, Franklin BA (2008). Physical activity and public health: updated recommendation for adults from the American College of Sports Medicine and the American Heart Association. Med Sci Sports Exerc.

[27] World Health Organization. Global recommendations on physical activity for health. Geneva World Health Organization. 2010;1–60. 26180873

[28] Otero M, Domínguez-Gil A (2000). Propuesta de un indicador de la “clase social” basado en la ocupación. Farm Hosp.

[29] Marrugat J, Solanas P, D’Agostino R, Sullivan L, Ordovas J, Cordón F (2003). Estimación del riesgo coronario en España mediante la ecuación de Framingham calibrada. Rev Española Cardiol.

[30] Ipaq. Guidelines for Data Processing and Analysis of the International Physical Activity Questionnaire ( IPAQ ) – Short and Long Forms. Ipaq. 2005;1–15.

[31] Rodríguez IT, Ballart JF, Pastor GC, Jordà EB, Val VA (2008). Validation of a short questionnaire on frequency of dietary intake: reproducibility and validity. Nutr Hosp.

[32] Arija V, Salas Salvado J, Fernández-Ballart J, Cuco GM-H (1996). Consumo alimentario, hábitos, y estado nutricional de la población de Reus (VIII). Evolución de la ingesta energética y nutricional desde 1983 a 1993. Med. Clin. (Barc).

[33] Rippe JM, Price JM, Hess SA, Kline G, DeMers KA, Damitz S (1998). Improved psychological well-being, quality of life, and health practices in moderately overweight women participating in a 12-week structured weight loss program. Obes Res.

[34] Ramos R, Solanas P, Cordón F, Rohlfs I, Elosua R, Sala J (2003). Comparación de la función de Framingham original y la calibrada del REGICOR en la predicción del riesgo coronario poblacional. Med. Clin. (Barc)..

[35] Hegde SM, Solomon SD (2015). Influence of physical activity on hypertension and cardiac structure and function. Curr Hypertens Rep.

[36] Besler C, Heinrich K, Rohrer L, Doerries C, Riwanto M, Shih DM (2011). Mechanisms underlying adverse effects of HDL on eNOS-activating pathways in patients with coronary artery disease. J Clin Invest.

[37] American Diabetes Association. Standards of Medical Care in Diabetes-2016. Diabetes Care. 2016;39 Suppl 1:S1–112. 15618112

[38] Segerström AB, Glans F, Eriksson K-F, Holmbäck AM, Groop L, Thorsson O (2010). Impact of exercise intensity and duration on insulin sensitivity in women with T2D. Eur J Intern Med.

[39] Myers J, Kaykha A, George S, Abella J, Zaheer N, Lear S (2004). Fitness versus physical activity patterns in predicting mortality in men. Am J Med.

[40] Mora S, Cook N, Buring JE, Ridker PM, Lee I-M (2007). Physical activity and reduced risk of cardiovascular events: potential mediating mechanisms. Circulation.

